# Intravitreal antisense oligonucleotide sepofarsen in Leber congenital amaurosis type 10: a phase 1b/2 trial

**DOI:** 10.1038/s41591-022-01755-w

**Published:** 2022-04-04

**Authors:** Stephen R. Russell, Arlene V. Drack, Artur V. Cideciyan, Samuel G. Jacobson, Bart P. Leroy, Caroline Van Cauwenbergh, Allen C. Ho, Alina V. Dumitrescu, Ian C. Han, Mitchell Martin, Wanda L. Pfeifer, Elliott H. Sohn, Jean Walshire, Alexandra V. Garafalo, Arun K. Krishnan, Christian A. Powers, Alexander Sumaroka, Alejandro J. Roman, Eva Vanhonsebrouck, Eltanara Jones, Fanny Nerinckx, Julie De Zaeytijd, Rob W. J. Collin, Carel Hoyng, Peter Adamson, Michael E. Cheetham, Michael R. Schwartz, Wilhelmina den Hollander, Friedrich Asmus, Gerard Platenburg, David Rodman, Aniz Girach

**Affiliations:** 1 University of Iowa Institute for Vision Research, University of Iowa, Iowa City, IA USA; 2 Department of Ophthalmology, Scheie Eye Institute, Perelman School of Medicine, University of Pennsylvania, Philadelphia, PA USA; 3 Center for Medical Genetics, Ghent University Hospital, Ghent, Belgium; 4 Department of Ophthalmology, Ghent University and Ghent University Hospital, Ghent, Belgium; 5 Division of Ophthalmology and Center for Cellular & Molecular Therapeutics, The Children’s Hospital of Philadelphia, Philadelphia, PA USA; 6 Wills Eye Hospital/Mid Atlantic Retina, Philadelphia, PA USA; 7 Department of Human Genetics and Donders Institute for Brain, Cognition and Behaviour, Radboud University Medical Center, Nijmegen, The Netherlands; 8 Department of Ophthalmology, Donders Institute for Brain, Cognition and Behaviour, Radboud University Medical Center, Nijmegen, The Netherlands; 9 UCL Institute of Ophthalmology, London, UK; 10 ProQR Therapeutics, Leiden, The Netherlands; 11 3D-PharmXchange, Tilburg, The Netherlands; 12 Oxular Limited, Magdalen Centre, Oxford, UK; 13 Mineralys Therapeutics, Radnor, PA USA

**Keywords:** Medical research, Genetics research, Translational research

## Abstract

CEP290-associated Leber congenital amaurosis type 10 (LCA10) is a retinal disease resulting in childhood blindness. Sepofarsen is an RNA antisense oligonucleotide targeting the c.2991+1655A>G variant in the *CEP290* gene to treat LCA10. In this open-label, phase 1b/2 (NCT03140969), 12-month, multicenter, multiple-dose, dose-escalation trial, six adult patients and five pediatric patients received ≤4 doses of intravitreal sepofarsen into the worse-seeing eye. The primary objective was to evaluate sepofarsen safety and tolerability via the frequency and severity of ocular adverse events (AEs); secondary objectives were to evaluate pharmacokinetics and efficacy via changes in functional outcomes. Six patients received sepofarsen 160 µg/80 µg, and five patients received sepofarsen 320 µg/160 µg. Ten of 11 (90.9%) patients developed ocular AEs in the treated eye (5/6 with 160 µg/80 µg; 5/5 with 320 µg/160 µg) versus one of 11 (9.1%) in the untreated eye; most were mild in severity and dose dependent. Eight patients developed cataracts, of which six (75.0%) were categorized as serious (2/3 with 160 µg/80 µg; 4/5 with 320 µg/160 µg), as lens replacement was required. As the 160-µg/80-µg group showed a better benefit–risk profile, higher doses were discontinued or not initiated. Statistically significant improvements in visual acuity and retinal sensitivity were reported (post hoc analysis). The manageable safety profile and improvements reported in this trial support the continuation of sepofarsen development.

## Abstract (web summary)

Intravitreal administration of the antisense oligonucleotide sepofarsen in patients with Leber congenital amaurosis type 10 showed a manageable safety profile, ocular adverse events being dose dependent, and achieved meaningful improvements of visual function.

## Main

Leber congenital amaurosis (LCA) is a group of inherited retinal diseases with an estimated prevalence of two per 100,000 individuals that affect both cone and rod photoreceptor cells from birth^1^. Variants in the centrosomal protein 290 (CEP290) gene cause LCA10 (refs. ^1,2^), a common and severe form of LCA, accounting for up to 30% of cases^3–7^ with an estimated prevalence of fewer than one patient per 100,000 individuals. LCA10 typically manifests as severe visual loss from early life^4,8,9^. The *CEP290* gene, located on chromosome 12 (ref. ^4^), encodes a 290-kDa centrosomal protein, involved in the formation and stability of the connecting cilium within retinal photoreceptor cells^10^ and other ciliated structures^11^. The c.2991+1655A>G variant in intron 26 of *CEP290* represents the most frequently occurring mutation in Europe and North America^5,10,12–14^. This variant introduces a cryptic splice donor site that causes insertion of an additional exon (pseudoexon) in *CEP290* mRNA, resulting in a premature stop codon (p.Cys998*), which leads to nonsense-mediated decay or truncated CEP290 protein formation^4,15–17^. The reduction in functional CEP290 protein is thought to disrupt protein trafficking between the cell body and the photoreceptor outer segment through the connecting cilium^18^ as well as to reduce both the cilium and outer segment formation^17,18^.

There are currently no treatments for LCA10. Novel therapeutic agents engineered to unique RNA target sequences, called antisense oligonucleotides (AONs), have been shown to provide meaningful clinical benefits^19–24^, including the first ‘N-of-1’ AON for Batten disease^22^. Between 2016 and 2020, eight different AONs received U.S. Food and Drug Administration (FDA) clearance to treat various diseases, including rare non-ocular genetic disorders^23^. Because of their targeting precision, relatively small size and chemically modified backbone with reduced degradation rates, AONs can provide therapeutic benefit and restore protein function in human diseases where disease variants of a gene are too large to be contained by adeno-associated virus 2 or other acceptable viral vectors for gene replacement or augmentation therapy. The most important mechanism of action of AONs is to silence either a splice site or a mutant sequence, resulting in exon skipping or variant silencing, respectively, at the mRNA level, a functionality that is challenging to achieve with current vector-delivered gene therapeutic agents. Unlike gene augmentation, which adds a functional copy of a missing or mutated gene^15^, sepofarsen, a 17-mer 2′-*O*-methyl-modified phosphorothioate antisense RNA oligonucleotide^24^, blocks splice donor sites that were erroneously created in the mRNA^15,25^. Sepofarsen binds to the deep intronic variant in the pseudoexon region and prevents recognition by splice factors, resulting in normal *CEP290* splicing of the pre-mRNA transcript and production of full-length CEP290 protein^15,17,24–26^.

This first-in-human trial assessed the safety, systemic pharmacokinetics and preliminary efficacy of intravitreally administered sepofarsen for the treatment of patients with LCA10 due to the c.2991+1655A>G variant. An interim analysis of the 3-month data and selected case study details, showing noticeable improvements in vision, were reported previously^25,27^, and here we report the final 12-month data^28^, including information on durability of response and longer-term safety follow-up.

## Results

### Patient disposition and baseline characteristics

**Extended Data Fig. 1:**
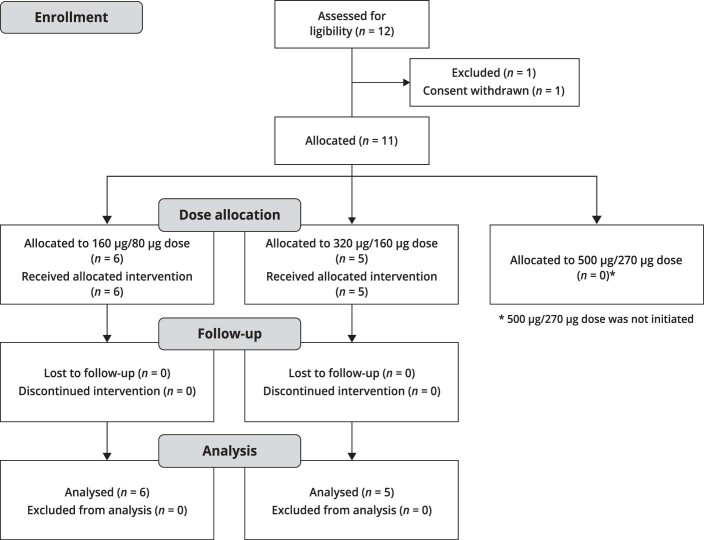
CONSORT trial flow diagram. During the enrollment period, 12 patients (6 adults and 6 children) were screened; 1 child withdrew consent before treatment. Of the 11 patients that enrolled, 6 received loading/maintenance doses of sepofarsen of 160µg/80µg and 5 received 320µg/160µg. All patients completed the full 12-month trial, and all were included in the analysis.

Participants were recruited between 8 November 2017 and 27 September 2018. Clinical data were collected at the University of Iowa Institute for Vision Research, University of Iowa, in Iowa City, Iowa; the Department of Ophthalmology, Scheie Eye Institute, Perelman School of Medicine, University of Pennsylvania, in Philadelphia, Pennsylvania; and Ghent University Hospital in Ghent, Belgium, between October 2017 and December 2019. During the enrollment period, 12 patients (six adults and six children) were screened; one child withdrew consent before treatment. Baseline characteristics are shown in Table 1. Of the 11 patients who enrolled (8–44 years of age), six received loading/maintenance doses of sepofarsen of 160 µg/80 µg, and five received 320 µg/160 µg (Extended Data Fig. 1). All 11 patients received a loading dose, whereas subsequent dosing frequency varied by patient (ranging from zero to three maintenance doses every 3–6 months) (Extended Data Table 1). All patients completed the full 12-month trial. The trial dose-escalation plan and dosing interval were adjusted, guided by the individual and study-level safety and efficacy data (see the safety section and Extended Data Table 1). Based on emerging safety, pharmacokinetic and pharmacodynamic data of the first two dose levels, the 500-µg/270-µg dose group was not initiated.

**Table 1:** Individual patient demographics and clinical characteristics

Patient identifier	Sex	Age, years	Second *CEP290* allele^a^	Second *CEP290* mutation protein annotation	Dose, µg^b^	Total number of doses received / Total cumulative dose over 12 months, µg	Baseline BCVA, logMAR	
							Treated eye	Untreated eye
P1	M	19	c.2506_2507del	p. Glu836Ilefs^a^2	160/80	4 / 400	4.0	4.0
P2	M	41	c.4723A>T	p.Lys1575^a^	160/80	3 / 320	4.0	4.0
P3	M	44	c.5668G>T	p.Gly1890^a^	160/80	3 / 320	2.4	2.3
P4	F	16	c.4438‐3del	NA	160/80	3 / 320	2.5	2.5
P5	M	8	c.6277del	p.Val2093Serfs^a^4	160/80	3 / 320	2.1	1.9
P6	F	21	c.3175dup	p.Ile1059Asnfs^a^11	320/160	3 / 640	4.0	4.0
P7	F	27	c.4723A>T	p.Lys1575^a^	320/160	3 / 640	1.1	0.7
P8	M	10	c.6277del	p.Val2093Serfs^a^4	320/160	2 / 480	1.9	1.4
P9	F	24	c.4393C>T	p.Arg1465^a^	320/160	1 / 320	4.0	4.0
P10	F	15	c.547_550del	p.Tyr183Argfs^a^4	320/160	2 / 480	4.0	4.0
P11	F	14	c.2991+1655A>G	p.Cys998^a^	160/80	1 / 160	0.6	0.6

The patient identifiers, variants and protein annotations presented in Table 1 correspond to the ones already reported in Cideciyan et al.^25^, and any differences reflect only changes in the nomenclature guidelines. The NCBI Reference Sequence identifier used for variant nomenclature is NM_025114. P11 was included in the trial after the 3-month interim analysis.

^a^All patients had c.2991+1655A>G/p.Cys998* allele in common; nucleotide change in the additional allele is shown.

^b^Loading/maintenance dose of sepofarsen was injected intravitreally in a 50-μl volume.

^c^A logMAR value of +4.0 was used in this trial to represent LP; this value places the category of LP in a roughly equidistant step, going from CF over detection of HM (corresponding to logMAR value of +2.0 and +3.0, respectively).

F, female; M, male.

### Safety, tolerability and dose adaptation

A summary of ocular adverse events is shown in Table 2. Ten of 11 (90.9%) patients developed ocular AEs in the treated eye versus one of 11 (9.1%) in the untreated eye; most were mild in severity and dose dependent. Eight patients developed cataracts in the treated eye (3/6 in the 160-µg/80-µg dose group and 5/5 in the 320-µg/160-µg dose group). Cataracts reported for the five patients in the 320-µg/160-µg dose group occurred earlier (from month 3 to month 9) than in the 160-µg/80-µg dose group (from month 8 to month 12) and were graded with a higher severity than the cataracts reported for the three patients in the 160-µg/80-µg dose group. Six cataracts were categorized as serious adverse events (SAEs), based on requirement of lens replacement; patients who showed a decline in visual acuity during cataract development regained their pre-cataract visual acuity (best-corrected visual acuity (BCVA)) after standard lens replacement surgery with no procedural complications. The only AE reported in the untreated eye was eye pruritus.

**Table 2:** Summary of ocular AEs

	Sepofarsen 160 μg/80 μg (*n* = 6)^a^	Sepofarsen 320 μg/160 μg (*n* = 5)^b^	Sepofarsen combined (*n* = 11)^c^	Relationship to study drug	Severity
Number of patients (%)					
**Ocular AE**	**5 (83**.**3)**	**5 (100)**	**10 (90**.**9)**		
**Treated eye**					
Conjunctival hemorrhage	4 (66.7)	4 (80.0)	8 (72.7)	Not related	Mild
Cataract^d^	3 (50.0)	5 (100)	8 (72.7)	Probable to	Mild to
SAEs	2 (33.0)	4 (80.0)	6 (54.5)	definite	severe
Conjunctival hyperemia	1 (16.7)	2 (40.0)	3 (27.3)	Not related	Mild
Dry eye	1 (16.7)	0	1 (9.1)	Not related	Mild
Retinal degeneration^e^	0	2 (40.0)	2 (18.2)	Probable	Mild to moderate
Anterior chamber cells	0	1 (20.0)	1 (9.1)	Not related	Mild
Conjunctival edema	0	1 (20.0)	1 (9.1)	Not related	Mild
Corneal lesion^f^	0	1 (20.0)	1 (9.1)	Not related	Mild
Cystoid macular edema	0	1 (20.0)	1 (9.1)	Probable	Mild
Diplopia	0	1 (20.0)	1 (9.1)	Definite	Moderate
Eyelid cyst	0	1 (20.0)	1 (9.1)	Not related	Mild
Eyelid edema	0	1 (20.0)	1 (9.1)	Not related	Mild
Hypotony of eye	0	1 (20.0)	1 (9.1)	Not related	Mild
Injection site hemorrhage	0	1 (20.0)	1 (9.1)	Not related	Mild
Lens disorder^g^	0	1 (20.0)	1 (9.1)	Possible	Mild
Metamorphopsia	0	1 (20.0)	1 (9.1)	Possible	Mild
Post-procedural complication^h^	0	1 (20.0)	1 (9.1)	Not related	Mild
Posterior capsule opacification	0	1 (20.0)	1 (9.1)	Not related	Mild
Retinal cyst^i^	0	1 (20.0)	1 (9.1)	Possible	Mild
Vitreal cells	0	1 (20.0)	1 (9.1)	Not related	Mild
Vitreous opacities	0	1 (20.0)	1 (9.1)	Possible	Mild
**Untreated eye**					
Eye pruritus	0	1 (20.0)	1 (9.1)	Not related	Mild

^a^Adults *n* = 3, children *n* = 3

^b^Adults *n* = 3, children *n* = 2

^c^Adults *n* = 6, children *n* = 5

^d^Cataract events comprised events coded as the following (number in 160-µg/80-µg dose group, number in 320-µg/160-µg dose group): cataracts (3, 4), cataract subcapsular (1, 0), cataract cortical (1, 0) and lenticular opacities (may represent an early stage of cataract development; 1, 3).

^e^Retinal degeneration comprised retinal thinning and retinal atrophy on optical coherence tomography imaging events.

^f^Corneal lesion corresponds to punctiform lesions in the cornea after lens replacement.

^g^Fewer than ten small round vacuoles in lens nucleus

^h^Air bubble in superior periphery of vitreous, after injection

^i^Retinal cyst event refers to the event of parafoveal intraretinal fluid cysts in the text (mild cystoid macular edema).

Based on incidence, severity and onset of cataracts, the decision was made, in consultation with the data monitoring committee, to discontinue dosing in the 320-µg/160-µg cohort and to apply a dosing interval of at least 6 months for the 160-µg/80-µg dose cohort in the second half of the trial.

Three patients in the 320-µg/160-µg dose group had retinal changes: one patient developed mild cystoid macular edema; one patient developed mild parafoveal intraretinal fluid cysts (macular edema) with retinal atrophy (considered as moderate retinal degeneration); and one patient showed retinal thinning (mild retinal degeneration). The cases of macular edema appeared to be resolving after month 12 in the first patient and at month 12 in the second patient upon treatment with either topical steroids or dorzolamide. No systemic therapy for either condition was required or administered. Retinal thinning/atrophy cases trended toward stabilization by month 12.

A summary of non-ocular AEs is shown in Extended Data Table 2. Non-ocular AEs were mostly of mild intensity or transient and not related to the study drug. There were no clinically relevant changes in clinical values, infrared imaging, laboratory variables, vital signs, physical examination or any of the electrocardiogram variables.

### Visual acuity

**Fig. 1:**
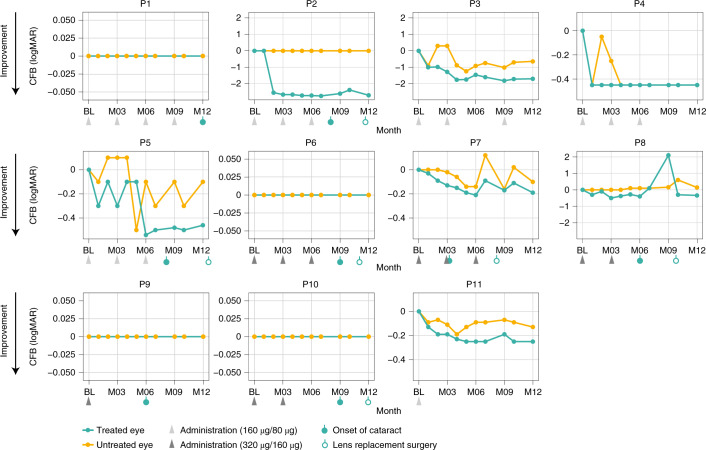
Change from baseline in BCVA at month 12 in individual patients (*n* = 11). Five of 11 patients (45%) showed a clinically meaningful improvement of at least −0.3 logMAR, and seven of 11 patients (64%) showed an improvement of at least −0.2 logMAR. P1, P2, P6, P9 and P10 were LP at baseline. Different scales of *y* axis are intended to facilitation data visualization. Eight patients developed cataracts in the treated eye (3/6 in the 160-µg/80-µg dose group and 5/5 in the 320-µg/160-µg dose group) at different time points during the trial (P7 at M3, P8 and P9 at M6, P2 and P5 at M8, P6 and P10 at M9 and P1 at M12). Six cataracts of eight needed lens replacement. These patients regained their pre-cataract visual acuity after surgery. For P8, from M7 onwards, the cataract quickly worsened, with post-capsular opacity grade 1 as well as a cortical opacity grade 2.5 reported at M9, which had substantial effect on BCVA, leading to a decision to perform lens replacement surgery a month later. P5 and P10 lens replacements were performed after M12. The pre-specified baseline (BL) was defined as the average pre-treatment value (that is, average of screening and day 1 (pre-dose) values). CFB, change from baseline; M, month.

**Fig. 2:**
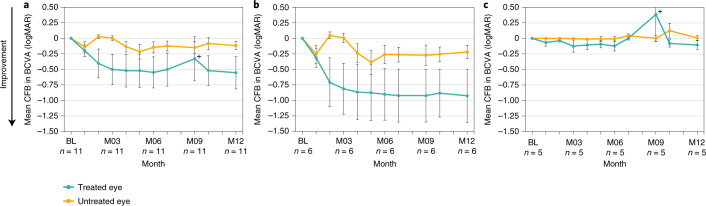
Mean change from baseline (CFB) to month 12 in BCVA comparing treated and untreated eyes. Treated and untreated eye groups are shown for the pooled dose groups (*n* = 11) (**a**); the low-dose (160-µg/80-µg) group (*n* = 6) (**b**); and the mid-dose (320-µg/160-µg) group (*n* = 5) (**c**). Bars show s.e.m.; data are summarized in Supplementary Table 1. +Visual acuity changes associated with cataract events occurrence: eight patients developed cataracts in the treated eye (3/6 in the 160-µg/80-µg dose group and 5/5 in the 320-µg/160-µg dose group), and six cataracts required lens replacement. These patients regained their pre-cataract visual acuity after surgery. BL, baseline; M, month.

For the pooled group (*n* = 11, including both dose levels), BCVA of treated eyes improved from baseline to month 12 with at least −0.3 logarithm of the minimum angle of resolution (logMAR) (gain of at least 15 letters on the Early Treatment Diabetic Retinopathy Study (ETDRS) vision chart) for five of the 11 patients (45%; *n* = 4 in the 160-µg/80-µg dose group, *n* = 1 in the 320-µg/160-µg dose group), compared to two of 11 in untreated eyes (18%; both in the 160-µg/80-µg dose group) (Fig. 1). For one patient (P4), both eyes improved similarly (−0.45 logMAR change). In the light perception (LP) population, four of five patients (80%) did not show BCVA changes at month 12. Of note is the −2.7 logMAR improvement observed in one LP patient, resulting in a logMAR value of +1.3 (Snellen equivalent 20/400) at month 12, enabling the patient to read letters on the ETDRS chart, and a −1.7 logMAR improvement observed in one hand motion patient, resulting in a logMAR value of +0.7 (Snellen equivalent 20/100) at month 12. BCVA improvements observed were generally maintained over a 12-month period, whereas the dosing interval was at least 6 months for most patients. For the one homozygous patient (P11), a patient with relatively good vision at baseline (+0.63 logMAR, 20/80 Snellen equivalent), the observed BCVA improvement of −0.25 logMAR (+13 ETDRS letters) could be sustained for at least 12 months after the initial 160-µg loading dose only (Fig. 1). In the pooled dose group, mean BCVA improvement from baseline to month 12 in treated eyes was −0.55 ± 0.862 logMAR (mean ± s.d., compared to −0.12 ± 0.228 logMAR in untreated eyes (*P* < 0.05)) (Fig. 2a and Supplementary Table 1). In the 160-µg/80-µg dose group (*n* = 6), this BCVA improvement was −0.93 ± 1.049 logMAR, compared to −0.22 ± 0.264 logMAR in untreated eyes (*P* = 0.13), whereas, in the 320-µg/160-µg dose group (*n* = 5), the change was −0.11 ± 0.155 logMAR, compared to 0.01 ± 0.086 logMAR in untreated eyes (*P* = 0.5) (Fig. 2b,c and Supplementary Table 1). Similar BCVA improvements were observed after a post hoc analysis using the best of BCVA pre-treatment values as baseline. In this analysis, one of 11 untreated eyes showed a −0.3 logMAR improvement, and the mean change from baseline at month 12 for all treated eyes was −0.45 ± 0.81 logMAR, compared to +0.03 ± 0.11 logMAR in untreated eyes (*P* = 0.031).

### Red and blue full-field stimulus test

**Fig. 3:**
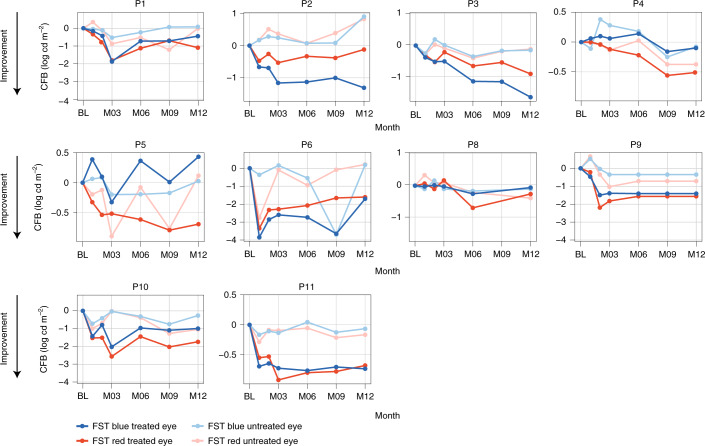
Change from baseline in red and blue FST at month 12 in treated and untreated eyes in individual patients (*n* = 10). Order of graphs and different scale of *y* axis are intended to facilitate data visualization. FST baseline data with the pulse stimulus test were missing for P7, so FST data for this patient were excluded from the efficacy analysis. For P9, M9 and M12 data were imputed using the last observation carried forward because FST was mistakenly tested with a flash stimulus instead of the pulse stimulus at these time points. BL, baseline; CFB, change from baseline; M, month.

**Fig. 4:**
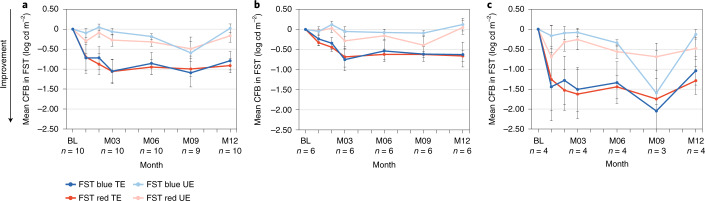
Mean change from baseline (CFB) to month 12 in blue and red FST comparing treated and untreated eye groups. Treated and untreated eye groups are shown for the pooled dose groups, *n* = 10 (**a**); the low-dose (160-µg/80-µg) group (*n* = 6) (**b**); and the mid-dose (320 µg/160 µg) group (*n* = 4) (**c**). Bars show s.e.m.; data are summarized in Supplementary Table 1. Enhanced FST response seemed most apparent in patients with visual acuity of light perception; three of five patients (60%) were LP at baseline in the mid-dose (320-µg/160-µg) group, and two of six patients (33%) were LP at baseline in the low-dose (160-µg/80-µg) group. BL, baseline; M, month; TE, treated eyes; UE, untreated eyes.

Improvements in red and blue full-field stimulus test (FST) from baseline to month 12 were observed in treated compared to untreated eyes (Fig. 3). In the pooled dose group (*n* = 10 (baseline FST value missing for one patient in the 320-µg/160-µg dose group)), improvements in red FST from baseline to month 12 were greater in treated eyes (mean ± s.d., −0.91 ± 0.571 logarithm of candela per square meter (log cd m^−^^2^)) than in untreated eyes (−0.16 ± 0.516 log cd m^−^^2^) (*P* < 0.01) (Fig. 4a). There was also greater improvement in blue FST from baseline to month 12 in treated eyes (−0.79 ± 0.731 log cd m^−^^2^) than in untreated eyes (+0.02 ± 0.347 log cd m^−^^2^) (*P* < 0.02).

In the 160-µg/80-µg dose group (*n* = 6), red FST improved from baseline to month 12 in treated eyes (−0.66 ± 0.332 log cd m^−^^2^) versus untreated eyes (+0.05 ± 0.417 log cd m^−^^2^) (*P* < 0.05), and blue FST showed a similar trend in treated eyes (−0.63 ± 0.757 log cd m^−^^2^) versus untreated eyes (+0.12 ± 0.392 log cd m^−^^2^) (*P* = 0.09) (Fig. 4b). In the 320-µg/160-µg dose group (*n* = 4), red and blue FST also improved from baseline to month 12 in treated eyes (−1.29 ± 0.693 log cd m^−^^2^ and −1.04 ± 0.717 log cd m^−^^2^, respectively) versus untreated eyes (−0.48 ± 0.535 log cd m^−^^2^ (nominal *P* = 0.25) and −0.13 ± 0.237 log cd m^−^^2^ (*P* = 0.25), respectively) (Fig. 4c).

### Mobility course

**Extended Data Fig. 2:**
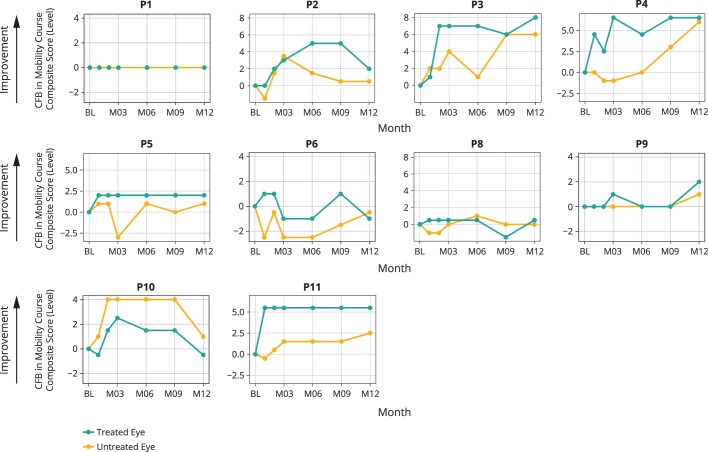
Change from baseline in mobility course composite score to Month 12 in treated and untreated eyes in individual patients (n = 10). Order of graphs and different scale of y-axis are intended to facilitation data visualization. P7 was not included in the mobility course composite score analysis because no baseline data were available. For this patient, at Month 12, the mobility course composite score revealed navigation to 19 of 20 levels for both treated and untreated eyes, suggesting no worsening from the treatment. BL = baseline; CFB = change from baseline; M = month; P = patient; TE = treated eye; UE = untreated eye.

In the pooled dose group (*n* = 10 (baseline mobility course composite score missing for one patient)), mean ± s.d. improvement in mobility course composite score was +2.50 ± 3.118 in treated eyes compared to +1.75 ± 2.383 in untreated eyes (*P* = 0.10). Improvements in the composite mobility course scores from baseline to month 12 were seen in the 160-µg/80-µg dose group (*n* = 6; +4.00 ± 3.114 and +2.67 ± 2.714 for treated and untreated eyes, respectively (*P* = 0.06)) and were greater than those in the 320-µg/160-µg dose group (*n* = 5; +0.25 ± 1.323 and +0.38 ± 0.750, respectively). A summary of individual patient responses in the composite mobility course scores from baseline to month 12 is shown in Extended Data Fig. 2.

### Anatomic endpoints

**Extended Data Fig. 3:**
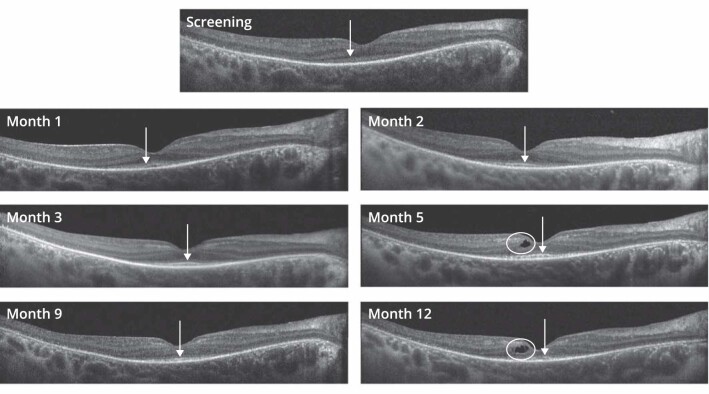
SD-OCT scans along the horizontal meridian crossing the fovea at screening, M1, M2, M3, M5, M9, and M12 for the patient P7. Arrows: the layer of the EZ line in all panels. Note that whereas the EZ line potentially shows improved definition post-treatment, it also shows some discontinuity at Month 12. Circles: cystoid macular edema in panels M5 and M12; retinal thinning in comparison of screening to M12. SD-OCT images of subject P7 provides examples of cystoid macular edema and retinal thinning. In this subject, these adverse events developed concurrent with reestablishment of the EZ, a feature of normal retinal architecture. SD-OCT = spectral-domain optical coherence tomography; M = month; EZ = ellipsoid zone.

For most patients, no meaningful changes were observed in the structural imaging endpoints via spectral domain optical coherence tomography (SD-OCT) and near-infrared autofluorescence (NIRAF) imaging due to the LCA10-related nystagmus that impeded duplication of the location of image capture. Structural changes reported as AEs in two patients are summarized in the safety section. One of those patients showed improved definition of the outer retinal bands, including the EZ band, after baseline and up to 9 months after treatment initiation. Thinning of the fovea and perifoveal regions, primarily of the outer nuclear layer, was seen at 12 months after treatment, albeit without visual change (Extended Data Fig. 3).

### Pharmacokinetics of sepofarsen

Sepofarsen serum levels were below the limit of quantitation (1.02 ng ml^−1^) after intravitreal administration of up to four doses in all patients at all time points, except on one occasion in one patient. This patient had sepofarsen levels of 1.53 ng ml^−1^ 3 hours after receiving the loading dose of 320 μg but not at any time point thereafter.

### Patient-reported outcomes

Visual Function Questionnaire-25 (VFQ-25) raw scores ranged from 0 to 100, with 100 representing the best and 0 representing the worst visual functioning. No generally accepted clinically relevant threshold has been defined for VFQ-25 in patients with a phenotype characteristic for LCA. Improvements from baseline in the total VFQ-25 score were observed at month 6; median change from baseline in the total VFQ-25 score was 8.6 for all adult patients. In terms of the VFQ-25 subscales, the most notable improvements at month 6, regardless of dose, were observed in the near vision and social function subscales. At month 12, median change from baseline in the total VFQ score was 3.1 for all patients. Safety events (retinal findings and cataracts) occurring between months 6 and 12 may have affected the degree of improvement observed at month 12. The small sample size, as well as the unknown definition of a clinically meaningful difference for the VFQ-25 score in patients with LCA, makes it difficult to interpret these data.

The total Cardiff Visual Ability Questionnaire for Children (CVAQC) score was presented in logit units on a scale from –2.96 to 2.80 (with lower scores indicative of better status). Pre-defined or exploratory thresholds were not defined for the CVAQC. The median change from baseline in total CVAQC score for all patients was 0.43 at month 6 (range, –0.53 to 0.72) and 0.54 at month 12 (range, –1.30 to 1.18). As with the VFQ-25 data, the small sample size made it difficult to interpret these data.

### Pupillary light reflex

In both eyes, mean changes from baseline in pupillary light reflex (PLR) maximum amplitude at 4,000 cd m^−^^2^ were generally small throughout the study, ranging from –0.2 ± 0.45 mm (month 3) to 0.0 mm (± 0.29 mm, month 1; ± 0.55 mm, month 12) in the treatment eye and 0.0 ± 0.54 mm (month 1) to 0.2 mm (± 0.54 mm, month 2; ± 0.61 mm, month 6; ±0.34 mm, month 12) in the contralateral eye. At month 12, mean changes from baseline in PLR maximum amplitude at 4,000 cd m^−^^2^ in the treatment eye were 0.0 ± 0.6 mm (treatment eyes of the pooled dose group), –0.1 ± 0.2 mm (160/80 µg) and 0.1 ± 0.9 mm (320/160 μg). Corresponding values in the contralateral eye were 0.2 ± 0.3 mm, 0.2 ± 0.4 mm and 0.1 ± 0.3 mm, respectively.

In both eyes, mean changes from baseline in PLR pre-stimulus at 4,000 cd m^−^^2^ were generally small throughout the study, ranging from –0.3 ± 0.86 mm (month 9) to 0.4 ± 0.90 mm (month 1) in the treatment eye and from –0.2 ± 0.56 mm (month 9) to 0.5 ± 0.70 mm (month 2) in the contralateral eye. At month 12, mean changes from baseline in PLR pre-stimulus at 4,000 cd m^−^^2^ in the treatment eye were 0.0 ± 0.7 mm (treatment eyes of the pooled dose group), 0.3 ± 0.6 mm (160/80 µg) and –0.4 ± 0.6 mm (320/160 μg). Corresponding values in the contralateral eye were 0.3 ± 0.9 mm, 0.5 ± 0.9 mm and 0.0 ± 0.7 mm, respectively.

### Other exploratory efficacy evaluations

An end-of-study electroretinogram (ERG) was required if a patient achieved one or more BCVA improvements of ≥15-letter improvement or progression from counting fingers (CF)/hand motion (HM) to logMAR +1.6 or progression from LP to CF/HM or a clinically meaningful improvement in at least one measure of visual function or retinal structure, in the opinion of the investigator. ERG results were uninterpretable, which was expected, due to an absence of biological signal and noise/artifacts, mainly coming from patients’ nystagmus as part of their background medical condition (LCA). None of the patients had a measurable signal at 12 months.

In both eyes, mean changes from baseline in oculomotor instability were small throughout the study.

Additional exploratory assessments (for example, visual evoked potential, color vision test and visual field test) could have been completed at the investigator’s discretion at any visit, and there are not enough data to interpret the results.

## Discussion

Here we report the results from a first-in-human trial in LCA10 due to the c.2991+1655A>G variant. Sepofarsen was generally well tolerated with a manageable safety profile and showed clinically relevant improvement in visual acuity, supported by improvements in retinal sensitivity (FST) and the ability to navigate a mobility course.

AONs have been used to target a variety of non-ocular genetic disorders. To date, the primary AON in ocular use is fomivirsen, to treat cytomegalovirus retinitis^23^. Sepofarsen is the first treatment to show a therapeutic benefit in LCA10, a retinopathy with generally severe visual impairment in which only a minority of patients reaching adulthood retain measurable vision. Therefore, sepofarsen has the potential to address a large unmet medical need in LCA10.

In this trial, sepofarsen resulted in a clinically meaningful gain of at least 15 letters in visual acuity (BCVA) on the ETDRS chart, in almost half of the patients treated. Visual acuity gains were generally corroborated by improvements in photoreceptor function (FST) and in the ability to navigate a mobility test. The final 12-month results presented here were consistent with the interim results reported previously^25,27^ and show that the initial therapeutic benefit observed within 3 months of treatment was maintained throughout the 12-month follow-up period. Minimal improvements were also observed in untreated eyes for some patients. Learning effects, motivation and variability may have contributed to these results in patients with very low vision, as the hypothesis of having a potential transport through the chiasm is highly unlikely^29^. Using the untreated eye as a control can be regarded as a conservative approach for assessing the effect size of sepofarsen in this first-in-human trial. Notably, statistically significant differences in treated versus untreated eyes were observed for BCVA and FST in the pooled group (including both dose groups) as well as the 160-µg/80-µg group, supporting the therapeutic potential of sepofarsen in LCA10. One patient showed a remarkable BCVA response at month 12 (an improvement of −2.7 logMAR). Even when these patient data were censored, the mean effect on BCVA was still maintained at clinically meaningful values in the aggregated treated eyes compared to untreated eyes.

In this investigational program for a novel AON, AEs reported as cataracts in the treated eyes were noted and could potentially be related to the interaction of sepofarsen with proteins or fibers in the lens. This hypothesis would require further analysis on the lens material removed from patients who developed cataracts. In addition, the incidence of cataracts is higher for patients with retinitis pigmentosa compared to the general population^30,31^, and the intravitreal injection procedure itself can also be related to cataract formation because of quiescent lens injury^32^, although lens injury was not observed within this trial. Notably, BCVA improvements pre-cataract were regained after cataract extraction and subsequent lens replacement surgery. Retinal changes reported were mild cystoid macular edema and retinal thinning/atrophy, neither associated with vision loss. These safety findings appeared to be dose dependent in this small study and led to the decision to prolong the dosing interval to at least 6 months and to discontinue dosing in the 320-µg/160-µg group. Despite these dose modifications, which reduced the number of doses in the latter half of the trial, the therapeutic benefit over a 12-month follow-up period was maintained, providing strong support for a dose interval longer than 3 months.

No biomarkers to determine patient response to sepofarsen were found; the only predictor of response identified was baseline visual acuity. One LP patient presented a substantial BCVA response (that is, the high responder showing a visual acuity improvement from LP to logMAR 1.3 (20/4,000)). An explanation may be related to the known history of measurable visual acuity during early childhood for this patient. For the other four patients, LP vision from birth could have negatively affected development of a functional visual cortex and could explain improvement in photoreceptor function as measured by FST without visual acuity improvement.

Of note, one patient homozygous for the c.2991+1655A>G variant with relatively good visual acuity at baseline (that is, Snellen acuity of 20/80) received just a single dose of 160-µg sepofarsen and showed maintained improvement in BCVA, FST and mobility course composite score through month 12 and beyond^27^. Molecularly, it can be hypothesized that the homozygous state, due to two sepofarsen-targetable alleles^24^, may be linked to a higher pre-mRNA correction rate and, therefore, a potentially longer therapeutic benefit from sepofarsen compared to that for heterozygous patients.

Limitations of this first-in-human trial in this ultra-rare indication include the small sample size, the open-label design, the absence of a sham-controlled group and the unequal distribution of LP patients (33% in the 160-µg/80-µg group versus 60% in the 320-µg/160-µg group). However, the efficacy data suggest a strong signal of visual improvement in treated versus untreated eyes, which was shown to be statistically significant.

The data reported here strengthen available preclinical data and provide clinical confirmation for the molecular mode of action of sepofarsen. Previously, concentration-dependent restoration of mRNA and protein profiles in LCA10 patient-derived fibroblasts and optic cups carrying the c.2991+1655A>G variant were reported^17,24,26^. In addition, studies in mice and rabbits showed that, on intravitreal injection, sepofarsen reaches the target area, the retina (in particular, the outer nuclear layer), and has a long half-life supportive for maintenance of efficacious levels and allowing infrequent dosing in the clinic.

Use of AONs as therapeutic agents targeting RNA is a growing area of investigation. Another AON that provides therapeutic benefits, nusinersen, was cleared for use by the FDA in December 2016 to treat spinal muscular atrophy types I through III^33^. In contrast to the systemic or intrathecal administration required for non-ocular diseases, a local intravitreal administration for retinal diseases, such as LCA10, has fundamental advantages because only relatively small doses of intrinsically long-acting AONs are required. Also, AONs have the potential to provide personalized treatment in rare and ultra-rare conditions, which was recently evidenced by the development of a systemic AON therapy for a single patient with a *CLN7* variant in Batten’s disease^22^.

As expected, the typical systemic safety concerns of AONs, which include inhibition of coagulation, activation of complement and accumulation in the liver, kidney and lymph nodes, were not observed with sepofarsen^23^. Of the 11 patients in this phase 1b/2 trial, nine enrolled into an extension trial to provide long-term data (NCT03913130, Insight trial). In addition, a phase 2b/3 trial is ongoing with the 160-µg/80-µg dose of sepofarsen. This trial has a 6-month dosing in the maintenance phase after month 3 (NCT03913143, Illuminate trial) and should provide further information on the ability of sepofarsen to restore some visual function in patients with LCA10 due to the c.2991+1655A>G variant in the *CEP290* gene.

## Methods

### Participants

Male or female patients, at least 6 years of age, with LCA10 due to biallelic variants in the *CEP290* gene, of which at least one was the c.2991+1655A>G variant, were eligible for enrollment. Eligible patients had a BCVA between LP (assigned to logMAR +4.0) and logMAR +0.6 (20/80 Snellen equivalent) in the worse-seeing eye and logMAR +0.4 (20/50 Snellen equivalent) or worse in the better-seeing eye, a detectable outer nuclear layer in the macular area by SD-OCT. Additional inclusion criteria included an ERG result consistent with LCA, clear ocular media and adequate pupillary dilation to facilitate retinal imaging. Exclusion criteria included syndromic disease, such as Joubert syndrome and Joubert syndrome-related disorders; any contraindication to intravitreal injection; pregnancy or breastfeeding; any severe renal or cardiac disease/defect; personal or family history of prolonged QT syndrome; abnormal laboratory findings; any ocular disease/condition that could compromise treatment safety or BCVA or interfere with assessment; any medical or psychiatric condition that could place the patient at undue risk or interfere with assessment; history of malignancy within 5 years before screening; intraocular surgery or intravitreal injection within 3 months before trial start; use of any investigational drug or device within 90 days or five half-lives before trial start; and prior gene therapy for LCA.

### Trial design and intervention

In this 12-month, multicenter, open-label, multiple-dose, dose-escalation, phase 1b/2 trial (PQ-110-001; ClinicalTrials.gov identifier NCT03140969), patients received sepofarsen (QR-110; ProQR Therapeutics) into the worse-seeing eye (treated eye) by intravitreal injection following standard procedures based on published guidelines^34^. The better-seeing eye was not treated (untreated eye).

**Extended Data Fig. 4:**
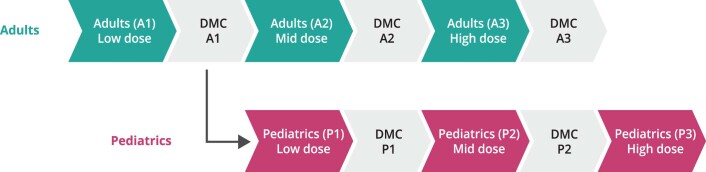
PQ-110-001 trial: Schema for introduction of dose levels. The safety monitoring committee and/or Sponsor Medical Monitor could decide to de-escalate the dose, hold the dose (delay or skip), or discontinue study drug for an individual subject, in consultation with the Investigator. Subjects who discontinued study drug continued to be followed for safety and efficacy. A = adult subjects; DMC = Data Monitoring Committee; P = pediatric subjects.

Selection of dose levels and interval between each dose were modeled based on the results of preclinical studies^24^. Up to three dose levels of sepofarsen were planned: 160 µg/80 µg, 320 µg/160 µg and 500 µg/270 µg for the loading/maintenance doses, respectively. Extended Data Fig. 4 summarizes the order and process for how the dose levels were selected. In each participant, up to four doses were to be administered at 3-month intervals through month 9. The final evaluation visit was scheduled for month 12.

The trial received institutional review board/ethics committee approval of the University of Iowa Institute for Vision Research, the University of Pennsylvania and the Ghent University Hospital. The trial was conducted in accordance with the ethical principles of Good Clinical Practice and the Declaration of Helsinki. Adult participants provided written informed consent. Age-appropriate assent and permission from the parent or legal guardian were obtained for children.

### Endpoints

The primary endpoint was safety and tolerability of sepofarsen, assessed by frequency and severity of ocular AEs in treated and untreated eyes at all study visits. Secondary endpoints included frequency and severity of non-ocular AEs; changes in ophthalmic examination findings; change from baseline in BCVA (assessed by the ETDRS vision chart or the Berkeley Rudimentary Vision Test (BRVT) for patients not able to read the letters on the ETDRS chart; Supplementary Table 2); change from baseline in dark-adapted retinal sensitivity using FST to red and blue stimuli; anatomic measurements such as change from baseline in retinal structural evaluations using SD-OCT and NIRAF; serum pharmacokinetic profile of sepofarsen; and change in amplitude and latency to white PLR. Ophthalmic assessments were performed at all study visits (Supplementary Table 3). An independent masked central reading center (EyeKor Inc.) was used to assess FST-, SD-OCT- and NIRAF-based endpoints. The serum pharmacokinetic profile of sepofarsen was assessed after intravitreal injection. Change from baseline in mobility course composite score was an exploratory endpoint and was interpreted and graded by a masked reader at Ora Inc. Additional exploratory endpoints included change in ocular instability; change in ERG; change in VFQ-25/CVAQC score; and change in biomarkers. Additional exploratory endpoints were included at the investigator’s discretion (change in light sensitivity to white FST; change in amplitude/latency to red/blue PLR; change in outer segment layer thickness by OCT; visual evoked potential; standard FST; standard color vision test; visual field tests; and additional light intensity tests on mobility course assessment).

#### Clinical assessments

Safety of sepofarsen was assessed by frequency and severity of ocular and non-ocular AEs in treated and untreated eyes at all study visits. AEs were coded using the Medical Dictionary for Regulatory Activities version 20.0. Severity of lens opacity was specifically assessed using the Age-Related Eye Diseases Study (AREDS) Clinical Lens Grading System (ARLNS).

BCVA was assessed at screening, day 1 (D1, pre-dose), month 1 (M1), M2, M3, M4, M5, M6, M7, M9, M10 and M12, using the ETDRS vision chart for patients able to read letters and the BRVT for patients not able to read letters. SD-OCT images were evaluated at screening and at M1, M2, M3, M4, M5, M6, M7, M9, M10 and M12 to detect structural changes, including thickness of the photoreceptor outer segment layer. The schedule of study visits and the assessments performed at each visit are presented in Supplementary Table 3.

#### Pharmacokinetic assessment

Samples for pharmacokinetic analysis were taken pre-dose and 3 hours post-dose on D1 and at the end of the study in all patients, although initially a more extended sampling scheme was applied in some patients (that is, pre-dose on all four dosing occasions; 3 hours and 18 hours, 7 days, 1 month and 2 months after the first dose; 7 days after the second dose; and final samples at the end of the study (12 months and 3–12 months after study drug administration)).

#### Visual acuity methods

The logMAR visual acuity was assessed using an ETDRS chart for each visit. For those unable to see a single letter on the ETDRS chart, the BRVT was performed. LP and HM were also acceptable measures of visual acuity for this trial for a patient unable to perform on the ETDRS chart and the BRVT. A BCVA equivalency table was used (Supplementary Table 2).

The observed value and change from baseline visual acuity was summarized for each eye (treated eye and untreated eye) for BCVA using continuous descriptive statistics on the logMAR scale by visit for each dose group and for all patients. logMAR was evaluated by either the ETDRS chart or the BRVT when the patient could not read the chart. A logMAR value of +4.0 was used in this trial to represent LP; this value places the category of LP in a roughly equidistant step, going from CF over detection of HM, corresponding to a logMAR value of +2.0 and +3.0, respectively^35–45^.

#### FST methods

Sensitivity to blue-light or red-light flashes presented in dark-adapted eyes was measured with FST because this test has a large dynamic range; it measures the full visual field, so it does not require fixation (patients with LCA typically have nystagmus)^46^. FST was tested with commercial hardware and software (Espion V6, Diagnosys)^47,48^. Each visit FST value was defined as the average of 8–20 values obtained per session. It was found that two patients were mistakenly tested at some visits (screening until month 2 for P7 and at month 9 and month 12 for P9) with a flash stimulus (duration, <4 ms) instead of the pulse stimulus (duration, 200 ms) specified in the protocol. FST baseline data with the pulse stimulus test were missing for P7, so FST data for this patient were excluded from the efficacy analysis, whereas FST data at month 9 and month 12 were imputed for P9 by replicating month 6 results.

#### SD-OCT

SD-OCT measured full retinal thickness, outer nuclear layer thickness, presence of macular edema and photoreceptor outer segment layer thickness. The schedule of study visits and the assessments performed at each visit are presented in Supplementary Table 3. Of note, due to severe nystagmus in most of the patients, it proved very difficult to acquire images of suitable quality to be able to accurately measure all of these parameters.

#### Mobility course methods

Mobility course testing was performed to assess functional vision by testing the ability of a patient to navigate a course accurately and at a reasonable pace at several levels of environmental illumination. There were four different mobility courses, each with a range of difficulty determined by different light levels: the Low-Contrast Visual Navigation Course, the High-Contrast Visual Navigation Course, the High-Contrast Room Exit and the Backlit Room Exit. Each course was designed for a certain visual impairment, with the most difficult course designed to be appropriate for patients with better vision (up to logMAR 0.4) and the least difficult course designed to be appropriate for patients with LP vision. Three separate mobility courses were initially included in the trial. The mobility course composite scores ranged from 0 to 12. A subset of patients was able to pass the most difficult light level available on the initial three courses included, requiring the addition of a fourth course of maximum difficulty after the start of the trial. The mobility course composite scores resulted in an updated composite scoring system ranging from 0 to 19. Higher mobility course composite scores are indicative of better status.

The order of testing on the mobility test begins at dimmer light levels; if the patient fails the test, the light level is increased until a pass is achieved. The most difficult course and light level combination passed yields the mobility course composite score, with each eye (treated eye, untreated eye and both eyes) graded independently. After a passing score is achieved for each eye, the test is concluded, because the most difficult course and light level combination passed have been identified. The site performed the initial grading, and an independent reading center (Ora Inc.) retrospectively performed final grading in a masked manner. The final course composite score was determined by the reading center. The mobility course composite score reflects the most difficult course and light level combination that patients could pass, as determined by the central grading center.

#### PLR methods

Patients with LCA10 have abnormalities in pupil reactions. Therefore, evaluation of pupil reactions via PLR was included as part of the study assessments. PLR imaging measures pupil size with various candela per square meter stimuli (that is, at 4, 40, 400 and 4,000 cd m^−2^); it is used to test integrity of the sensory and motor functions of the eye. A pre-defined threshold was not defined for PLR (that is, maximum amplitude minus pre-stimulus at 4,000 cd m^−2^). A best discriminating (treatment versus contralateral) threshold for changes from baseline was determined by receiver operator characteristic analysis over all post-baseline time points using the Youden index. For PLR amplitude parameters, higher values are indicative of better status. For PLR latency parameters, lower values are indicative of better status.

#### Patient-reported outcome methods

The National Eye Institute Visual Function Questionnaire is a questionnaire that measures dimensions of vision-targeted health status that are most important for people with chronic eye diseases. The survey measures the influence of visual disability on general health domains, such as emotional well-being and social functioning, in addition to task-oriented domains related to daily visual functioning in adult patients. This assessment tool has been validated and is the standard for ophthalmic disease studies. The CVAQC is a short, psychometrically robust and self-reported instrument for the assessment of visual ability in children with a visual impairment^49^. Pre-defined thresholds were not defined for these questionnaires within this specific patient population.

### Statistical analysis

For an ocular orphan disease, a sample size of approximately 12 patients was considered adequate to fulfill the safety objectives of the trial. Safety was analyzed in all patients who received at least one dose of sepofarsen. Efficacy was assessed in all patients who received at least one dose of sepofarsen and had at least one baseline and one post-baseline efficacy measurement. The pre-specified baseline was defined as the average pre-treatment value (that is, average of screening and day 1 (pre-dose) values); however, a post hoc analysis using the best pre-treatment BCVA at baseline, a more conservative analysis currently used for the phase 2b/3 trial, was also performed, and the related *P* value is reported as post hoc. Although efficacy endpoint analyses were exploratory, and no formal power analysis was performed, nominal two-sided *P* values for the Wilcoxon signed-rank test with no multiplicity control applied were reported to provide an assessment of the signal-to-noise ratio comparing the within-subject changes from baseline of treated eyes (regardless of dose) against those of untreated eyes. Descriptive analyses, mean, standard deviation and responder analyses are also reported. Data collection was performed with Spectralis 6.8 (Heidelberg Engineering) and Espion V6 (Diagnosys) software. Data analysis was performed with SAS 9.4 software. No custom code or algorithms were used.

### Reporting Summary

Further information on research design is available in the Nature Research Reporting Summary linked to this article.

## Online content

Any methods, additional references, Nature Research reporting summaries, source data, extended data, supplementary information, acknowledgements, peer review information; details of author contributions and competing interests; and statements of data and code availability are available at 10.1038/s41591-022-01755-w.

### Supplementary information

Supplementary Information Supplementary Information includes the list of investigators and Supplementary Tables 1–3

Reporting Summary

## Figures and Tables

**Extended Data Table 1:**
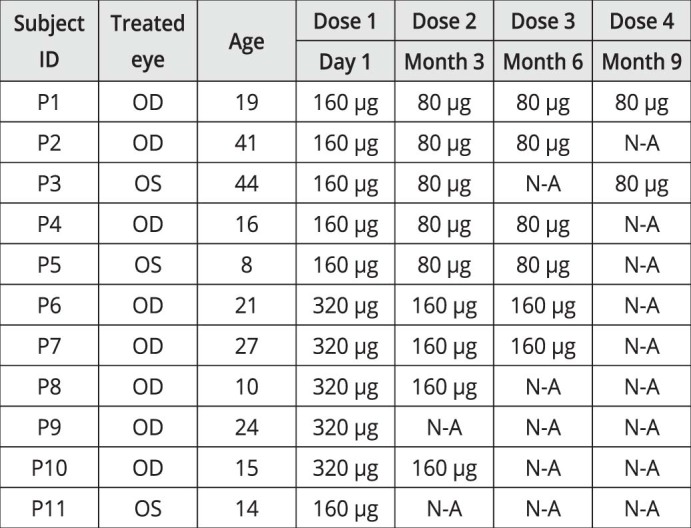
Study drug administration in trial PQ-110-001

Based on the benefit–risk from the first two dose levels, the decision was made, in consultation with the data monitoring committee, to not initiate the 500-µg/270-µg dose group. A dosing interval of at least 6 months for the 160-µg/80-µg dose cohort was also applied, and the 320-µg/160-µg cohort was discontinued. NA, dose not administered; OD, right eye; OS, left eye.

**Extended Data Table 2:**
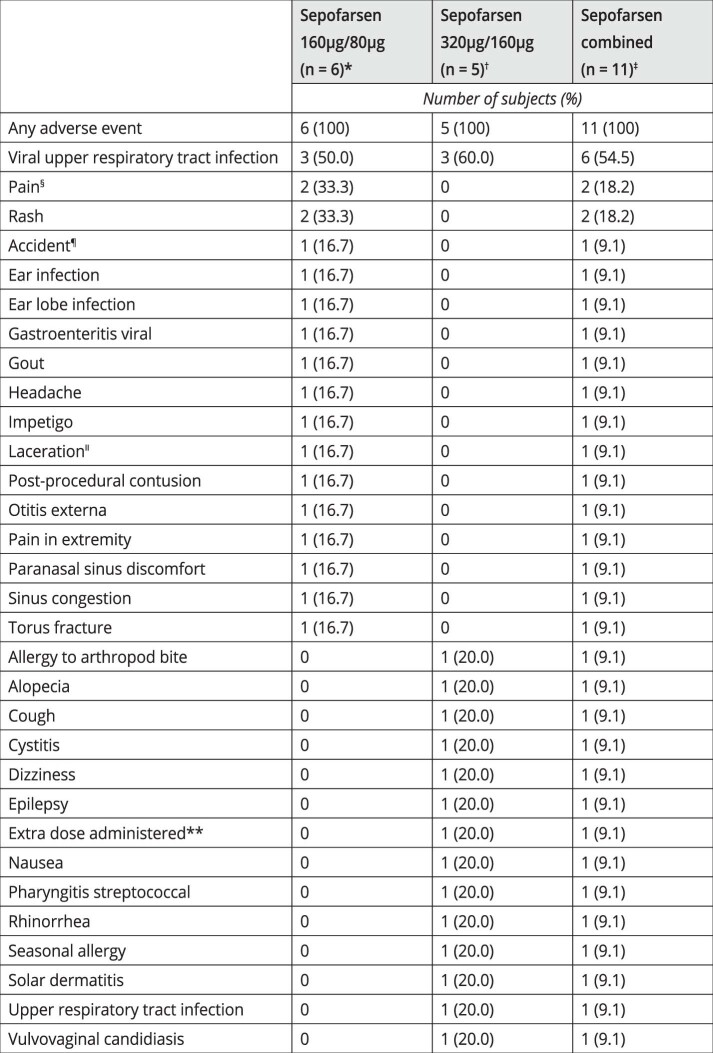
Summary of non-ocular AEs

*Adults *n* = 3, children *n* = 3. †Adults *n* = 3, children *n* = 2. ‡Adults *n* = 6, children *n* = 5. §Pain comprised whole body pain for one patient; mild body pain and paranasal sinus discomfort were reported in a second patient. ¶An accident with a belt sander was reported. ‖Severe laceration of the fifth finger of the right hand was reported and linked to the accident and not related to the study drug. **A double dose of anti-epileptic drug was administered.

## Data Availability

The database-approved official symbols used in this trial for the *CEP290* gene were the HUGO Gene Nomenclature Committee (https://www.genenames.org/data/gene-symbol-report/#!/hgnc_id/HGNC:29021) and National Center of Biotechnology Information Gene (https://www.ncbi.nlm.nih.gov/gene/80184). Some data that support the findings of this study are available from ProQR Therapeutics. However, restrictions apply to the availability of these data, including intellectual property and confidentiality obligations, and, so, they are not publicly available. Anonymized derived data will be made available by the authors upon reasonable request with a methodologically sound proposal and with the permission of ProQR Therapeutics at hcp@proqr.com. All requests for raw and analyzed data are promptly reviewed, within 2 months, by a ProQR Therapeutics delegate and trial organizer, to verify if the request is subject to any intellectual property or confidentiality restrictions. Patient-related data not included in the paper were generated as part of clinical trials and may be subject to patient confidentiality. Any data that can be shared will be released via a data use agreement.
