# Supplementary material for: Intravitreal antisense oligonucleotide sepofarsen in Leber congenital amaurosis type 10: a phase 1b/2 trial
Source: Nat Med. 2022 Apr 4;28(5):1014–21. doi: 10.1038/s41591-022-01755-w (PMC9117145; doi:10.1038/s41591-022-01755-w)
Supplement: Supplementary file 1 — Supplementary Information includes the list of investigators and Supplementary Tables 1–3 [file 41591_2022_1755_MOESM1_ESM.pdf]

nature portfolio

https://doi.org/10.1038/s41591-022-01755-w

Supplementary information

Intravitreal antisense oligonucleotide sepofarsen in Leber congenital amaurosis type 10: a phase 1b/2 trial

In the format provided by the authors and unedited

Supplementary Information

Table of Contents

LIST OF INVESTIGATORS 2

Table S1. BCVA and FST efficacy end points 3

Table S2. Visual acuities equivalency table 7

Table S3. Schedule of visits and assessments 8

LIST OF INVESTIGATORS

Stephen R. Russell, MD; Arlene V. Drack, MD; The University of Iowa Institute for Vision Research, University of Iowa, Iowa City, Iowa.

Artur V. Cideciyan, PhD; Samuel G. Jacobson, MD; Department of Ophthalmology, Scheie Eye Institute, Perelman School of Medicine, University of Pennsylvania, Philadelphia, Pennsylvania.

Bart P. Leroy, MD, PhD; Ghent University Hospital, Ghent, Belgium.

Table S1. BCVA and FST efficacy end points

	All subjects (N = 11)		Sepofarsen 160 µg/80 µg (N = 6)		Sepofarsen 320 µg/160 µg (N = 5)	
	TE	UE	TE	UE	TE	UE
BCVA (logMAR)/baseline						
n	11	11	6	6	5	5
Mean (SD)	2.78 (1.286)	2.67 (1.388)	2.6 (1.274)	2.55 (1.295)	2.99 (1.415)	2.82 (1.635)
SEM	0.388	0.419	0.52	0.529	0.633	0.731
Median	2.45	2.45	2.42	2.38	4	4
BCVA (logMAR)/M03						
n	11	11	6	6	5	5
CFB mean (SD)	−0.5 (0.809)	0 (0.132)	−0.81 (1.007)	0.01 (0.187)	−0.13 (0.217)	0 (0.009)
SEM	0.244	0.04	0.411	0.076	0.097	0.004
CFB median	−0.19	0	−0.38	0	0	0
BCVA (logMAR)/M06						
n	11	11	6	6	5	5
CFB mean (SD)	−0.55 (0.836)	−0.15 (0.294)	−0.9 (1.019)	−0.26 (0.364)	−0.12 (0.18)	−0.01 (0.086)
SEM	0.252	0.089	0.416	0.148	0.081	0.038
CFB median	−0.25	0	−0.5	−0.1	0	0
BCVA (logMAR)/M09						
n	11	11	6	6	5	5
CFB mean (SD)	−0.33 (1.175)	−0.15 (0.326)	−0.92 (1.042)	−0.27 (0.402)	0.39 (0.961)	0 (0.113)
SEM	0.354	0.098	0.425	0.164	0.43	0.051
CFB median	−0.17	0	−0.46	−0.08	0	0

	All subjects (N = 11)		Sepofarsen 160 µg/80 µg (N = 6)		Sepofarsen 320 µg/160 µg (N = 5)	
	TE	UE	TE	UE	TE	UE
BCVA (logMAR)/M12						
n	11	11	6	6	5	5
CFB mean (SD)	−0.55 (0.862)	−0.12 (0.228)	−0.93 (1.049)	−0.22 (0.264)	−0.11 (0.155)	0.01 (0.086)
SEM	0.26	0.069	0.428	0.108	0.069	0.038
CFB median	−0.25	0	−0.46	−0.12	0	0
FST blue (log cd/m2)/Baseline						
n	10	10	6	6	4	4
Mean (SD)	−0.3 (1.521)	−0.29 (1.372)	−0.42 (1.6)	−0.37 (1.511)	−0.11 (1.611)	−0.16 (1.344)
SEM	0.481	0.434	0.653	0.617	0.805	0.672
Median	−0.57	−0.62	−0.97	−0.9	0.24	−0.08
FST blue (log cd/m2)/M03						
n	10	10	6	6	4	4
CFB Mean (SD)	−1.05 (0.905)	−0.06 (0.256)	−0.75 (0.683)	−0.05 (0.301)	−1.51 (1.106)	−0.08 (0.21)
SEM	0.286	0.081	0.279	0.123	0.553	0.105
CFB Median	−0.94	−0.07	−0.61	−0.06	−1.7	−0.07
FST blue (log cd/m2)/M06						
n	10	10	6	6	4	4
CFB Mean (SD)	−0.86 (0.873)	−0.18 (0.224)	−0.54 (0.639)	−0.08 (0.206)	−1.34 (1.045)	−0.34 (0.157)
SEM	0.276	0.071	0.261	0.084	0.522	0.079
CFB Median	−0.86	−0.21	−0.74	−0.07	−1.18	−0.33
FST blue (log cd/m2)/M09						

	All subjects (*N* = 11)		Sepofarsen 160 µg/80 µg (*N* = 6)		Sepofarsen 320 µg/160 µg (*N* = 5)	
	TE	UE	TE	UE	TE	UE
n	9	9	6	6	3	3
CFB Mean (SD)	−1.09 (1.062)	−0.59 (1.189)	−0.62 (0.452)	−0.09 (0.138)	−2.05 (1.395)	−1.6 (1.829)
SEM	0.354	0.396	0.185	0.056	0.805	1.056
CFB Median	−1	−0.17	−0.71	−0.14	−1.41	−0.75
FST blue (log cd/m2)/M12						
n	10	10	6	6	4	4
CFB Mean (SD)	−0.79 (0.731)	0.02 (0.347)	−0.63 (0.757)	0.12 (0.392)	−1.04 (0.717)	−0.13 (0.237)
SEM	0.231	0.11	0.309	0.16	0.358	0.118
CFB Median	−0.86	−0.07	−0.59	−0.02	−1.2	−0.18
FST red (log cd/m2)/Baseline						
n	10	10	6	6	4	4
Mean (SD)	−0.14 (1.635)	−0.21 (1.573)	−0.27 (1.796)	−0.33 (1.734)	0.06 (1.6)	−0.02 (1.529)
SEM	0.517	0.497	0.733	0.708	0.8	0.765
Median	−0.56	−0.71	−0.97	−0.89	0.43	0.08
FST red (log cd/m2)/M03						
n	10	10	6	6	4	4
CFB Mean (SD)	−1.06 (0.978)	−0.27 (0.477)	−0.68 (0.621)	−0.28 (0.5)	−1.62 (1.233)	−0.26 (0.515)
SEM	0.309	0.151	0.253	0.204	0.616	0.258
CFB Median	−0.72	−0.1	−0.52	−0.12	−2.05	−0.05
FST red (log cd/m2)/M06						
n	10	10	6	6	4	4

	All subjects (N = 11)		Sepofarsen 160 µg/80 µg (N = 6)		Sepofarsen 320 µg/160 µg (N = 5)	
	TE	UE	TE	UE	TE	UE
CFB Mean (SD)	−0.95 (0.591)	−0.32 (0.335)	−0.62 (0.327)	−0.16 (0.241)	−1.44 (0.575)	−0.56 (0.331)
SEM	0.187	0.106	0.134	0.098	0.287	0.166
CFB Median	−0.74	−0.29	−0.62	−0.07	−1.5	−0.55
FST red (log cd/m2)/M09						
n	9	9	6	6	3	3
CFB Mean (SD)	−1 (0.59)	−0.49 (0.546)	−0.62 (0.163)	−0.39 (0.548)	−1.75 (0.246)	−0.69 (0.592)
SEM	0.197	0.182	0.067	0.224	0.142	0.342
CFB Median	−0.78	−0.38	−0.63	−0.3	−1.65	−0.71
FST red (log cd/m2)/M12						
n	10	10	6	6	4	4
CFB Mean (SD)	−0.91 (0.571)	−0.16 (0.516)	−0.66 (0.332)	0.05 (0.417)	−1.29 (0.693)	−0.48 (0.535)
SEM	0.181	0.163	0.135	0.17	0.347	0.268
CFB Median	−0.79	−0.14	−0.69	−0.06	−1.58	−0.55

Baseline is defined as the last available measurements obtained during the screening period, before first Investigational Medicinal Product dose administration.

BCVA = best-corrected visual acuity; CFB = change from baseline; M = month; SD = standard deviation; SEM = standard error of the mean; TE = treated eye; UE = untreated eye.

Table S2. Visual acuities equivalency table

Visual acuity	Snellen notation	Decimal fraction = Monoyer scale	LogMAR value	Equivalence BRVT test	
**Light perception***			+4.0*		
**Tested with clinical assessment at 60 cm**				**Tested at 1 m**	**Tested at 25 cm**
**Hand motion***	20/20000	0.001	+3.0*		
	20/16000	0.00125	+2.9		GA 200 M
	20/10000	0.002	+2.7		GA 125 M
	20/8000	0.0025	+2.6		STE 100 M
	20/6300	0.0032	+2.5		GA 80 M
	20/5000	0.004	+2.4		STE 63 M
	20/4000	0.005	+2.3		GA 50 M
	20/3200	0.00625	+2.2		STE 40 M
**Counting fingers***	20/2000	0.010	+2.0*	STE 100 M	STE 25 M
	20/1200	0.016	+1.8	STE 63 M	
**Tested with ETDRS at 1 m**	20/800	0.025	+1.6	STE 40 M	
	20/600	0.03	+1.5		
	20/500	0.04	+1.4	STE 25 M	
	20/400	0.05	+1.3		
	20/320	0.06	+1.2		
	20/250	0.08	+1.1		
**Tested with ETDRS at 4 m**	20/200	0.10	+1.0		
	20/160	0.13	+0.9		
	20/125	0.16	+0.8		
	20/100	0.20	+0.7		
	20/80	0.25	+0.6		
	20/63	0.32	+0.5		
	20/50	0.40	+0.4		
	20/40	0.50	+0.3		
	20/32	0.63	+0.2		
	20/25	0.80	+0.1		
	20/20	1.00	0		
	20/16	1.25	-0.1		
	20/12.5	1.60	-0.2		
	20/10	2.00	-0.3		

*A logMAR value of +4.0 has been used in this trial to represent light perception; this value places the category of light perception in a roughly equidistant step, going from counting fingers over detection of hand motion (corresponding to logMAR value of +2.0 and +3.0, respectively, based on Holladay, 1997). Data included in the grey part refer to values obtained through the BRVT test.

BRVT = Berkeley Rudimentary Vision Test; BWD = black and white discrimination; ETDRS = Early Treatment of Diabetic Retinopathy Study eye chart; GA = grating acuity; logMAR = logarithm of the minimum angle of resolution; M = M-units (with 1 M equivalent to 1.45 millimeters, based on Bailey, 2012): STE = single tumbling E; WFP = white field projection.

Table S3. Schedule of visits and assessments

Visit Numbers	Screen 1	2	3	4	5	6	7	8	9	10	11	12	13	14	15	16	17	18	19	20	21	Unscheduled Visit
Visit (Visit Window)	Day -28 to -1	D1	D2	D7 (±3d)	D14[o] (±3d)	M1 (±7d)	M2 (±7d)	M3 (±10d)	M3 +1d	M3 +7d (±3d)	M4 (±7d)	M5 (±7d)	M6 (±10d)	M6 +1d	M6+7d (±3d)	M7 (±7d)	M9 (±10d)	M9 +1d	M9+7d (±3d)	M10 (±7d)	M12/EOS[q] (±10d)	
**Assessment**[a]																						
Informed consent/ parental permission	X																					
Pediatric assent	X																					
Demographics	X																					
Height and weight	X												X[k, r]								X[r]	
Review of eligibility criteria	X	X[k]																				
Medical and ocular history	X																					
Concomitant medications	X	X[k]	X	X	X	X	X	X[k]	X	X	X	X	X[k]	X	X	X	X[k]	X	X	X	X	X
Genetic analysis	X																					
AEs		X[k]	X	X	X	X	X	X[k]	X	X	X	X	X[k]	X	X	X	X[k]	X	X	X	X	X
12 lead ECG	X					X		X[k]					X[k]								X	
Ophthalmic exam[b]	X	X[k]	X	X		X	X	X[k]	X	X	X	X	X[k]	X	X	X	X[k]	X	X	X	X	X
BCVA	X[h]	X[k]				X	X	X[k]			X	X	X[k]			X	X[k]			X	X	
Physical exam	X[i]	X[k,l]	X[l]	X[l]		X[l]		X[k,l]	X[l]		X[l]		X[i,k]			X[l]	X[k,l]			X[l]	X[i]	
Urinalysis	X																					
Hematology	X		X	X		X		X[k]		X	X		X[k]		X	X	X[k]		X	X	X	
Serum chemistry	X		X	X		X		X[k]		X	X		X[k]		X	X	X[k]		X	X	X	
Vital signs	X	X[k]	X	X		X	X	X[k]	X	X	X	X	X[k]	X	X	X	X[k]	X	X	X	X	
Pregnancy test (WOCBP only)[c]	X	X						X					X				X				X	
Administration of study treatment[d]		X						X[p]					X[p]				X[p]					

Visit Numbers	Screen 1	2	3	4	5	6	7	8	9	10	11	12	13	14	15	16	17	18	19	20	21	Unscheduled Visit
**Visit (Visit Window)**	**Day -28 to -1**	**D1**	**D2**	**D7 (±3d)**	**D14[o] (±3d)**	**M1 (±7d)**	**M2 (±7d)**	**M3 (±10d)**	**M3 +1d**	**M3 +7d (±3d)**	**M4 (±7d)**	**M5 (±7d)**	**M6 (±10d)**	**M6 +1d**	**M6+7d (±3d)**	**M7 (±7d)**	**M9 (±10d)**	**M9 +1d**	**M9+7d (±3d)**	**M10 (±7d)**	**M12/EOS[q] (±10d)**	
**Assessment[a]**																						
Monitoring for safety of IVT injection		X[m]	X[m]	X[m]	X[o]			X[m]	X[m]	X[m]			X[m]	X[m]	X[m]		X[m]	X[m]	X[m]			
Monitoring of safety of sedation or anesthesia		X						X					X				X					
Blood draw for PK		X[k,n]																			X	
CH50 and INR	X		X	X		X		X[k]		X	X		X[k]		X	X	X[k]		X	X	X	
Specialized immunologic and inflammatory biomarkers[e]		X[k]				X	X	X[k]					X[k]				X[k]				X	X
Inflammatory markers: ESR and CRP	X		X	X		X		X[k]		X	X		X[k]		X	X	X[k]		X	X	X	
Infrared imaging	X[h]					X	X	X[k]			X	X	X[k]			X	X[k]			X	X	
ERG	X[h,j]																				X[s]	
FST	X[h]	X[k]				X	X	X[k]					X[k]				X[k]				X	
Mobility course	X[h]	X[k]				X	X	X[k]					X[k]				X[k]				X	
PLR	X[h]	X[k]				X	X	X[k]					X[k]				X[k]				X	
OCT	X[h]					X	X	X[k]			X	X	X[k]			X	X[k]			X	X	
Oculomotor instability	X[h]	X[k]						X[k]					X[k]				X[k]				X	
VFQ-25/CVAQC[f]		X[k]											X[k]								X	
Exploratory assessments[g]	X																					

a Study visits could have been conducted in multiple consecutive days instead of 1 full day, at the discretion of the Investigator.

b Ophthalmic exam included BCVA; anterior segment examination, including grading of anterior chamber inflammation according to the SUN Working Group Grading Scheme for Anterior Chamber Flare; IOP; clinical lens grading using the AREDS Clinical Lens Grading System (ARLNS); and posterior segment/fundus examination,

including grading inflammation in the vitreous using the NIH Grading Scale for Vitreous Haze. BCVA included refraction. As BCVA was not required at all time points, these components of the exam were listed separately to clarify timing.

c Pre-dose. Serum pregnancy test was required at Screening. Urine pregnancy tests were acceptable at all other time points for WOCBP.

d Instructions for administration were provided in a Study Reference Manual.

e Biomarker samples (blood) were to be collected at any study visit or unscheduled visit at which inflammation, pain or redness of the eye was present.

f Adult subjects (≥18 years of age) completed the VFQ-25. Pediatric subjects (<18 years of age) completed the CVAQC.

g In addition to the assessments already listed, exploratory assessments (eg, VEP, color vision test, visual field test) could have been completed at the Investigator's discretion at any visit.

h A repeat of individual efficacy assessments was required during the Day −28 to Day −1 Screening period, if the results were considered unreliable or of unacceptable quality by the Investigator or if the assessment was not able to be completed.

i Complete physical exam.

j Historic ERG results were acceptable for purposes of study eligibility.

k Pre-dose.

l Symptom-directed physical exam.

m Following the IVT injection, IOP and/or perfusion of the optic nerve head were monitored and managed appropriately. Subjects were monitored for increases in IOP and signs of inflammation and endophthalmitis during the post-injection period.

n Second PK blood draw was done 3 hours post-dose on Day 1 only (± 1 hour).

o This visit was a phone call from the site to the subject. No travel was required. The site collected concomitant medications and AEs and inquired as to the subject's health following the IVT injection via phone.

p For subjects who continued to be eligible for repeat dosing.

q EOS visit. Subjects may have been eligible for continued dosing and, if so, were to receive their next scheduled dose of drug under the extension study protocol. For subjects who terminated from the study early, the EOS visit was conducted 3 months after the subject's last dose of study treatment.

r Pediatric subjects only.

s An EOS ERG was required only if a subject had achieved one or more of the following criteria relative to Baseline at any study visit.

- BCVA improvement: At least 15 letters improvement or progression from CF/HM to logMAR + 1.6 or progression from LP to CF/HM
- A clinically significant improvement in at least one measure of visual function or retinal structure, in the opinion of the Investigator

AE = adverse event; AREDS = Age-Related Eye Diseases Study; ARLNS = Clinical Lens Grading System; BCVA = best-corrected visual acuity; CF/HM = counting fingers/hand motion; CH50 = C4, hemolytic complement 50; CRP = C-reactive protein; CVAQC = Cardiff Visual Ability Questionnaire for Children; d/D = day; ECG = electrocardiogram; EOS = end of study; ERG = electroretinogram; ESR = erythrocyte sedimentation rate; FST = full-field stimulus testing; INR = international normalized ratio; IOP = intraocular pressure; IVT = intravitreal; logMAR = logarithm of the minimum angle of resolution; LP = light perception; M = Month (30 days); NIH = National Institutes of Health; OCT = optical coherence tomography; PK = pharmacokinetic; PLR = pupillary light reflex; SUN = Standardization of Uveitis Nomenclature; VEP = visual evoked potential; VFQ-25 = Visual Function Questionnaire-25; WOCBP = women of childbearing potential
