# Supplementary material for: Intravitreal antisense oligonucleotide sepofarsen in Leber congenital amaurosis type 10: a phase 1b/2 trial
Source: Nat Med. 2022 Apr 4;28(5):1014–21. doi: 10.1038/s41591-022-01755-w (PMC9117145; doi:10.1038/s41591-022-01755-w)
Supplement: Supplementary file 2 — Reporting Summary [file 41591_2022_1755_MOESM2_ESM.pdf]

Corresponding author(s): Stephen R. Russell, MD

Last updated by author(s): Jan 21, 2022

# Reporting Summary

Nature Research wishes to improve the reproducibility of the work that we publish. This form provides structure for consistency and transparency in reporting. For further information on Nature Research policies, see our Editorial Policies and the Editorial Policy Checklist.

## Statistics

For all statistical analyses, confirm that the following items are present in the figure legend, table legend, main text, or Methods section.

| n/a | Confirmed | |
|---|---|---|
| ☐ | ☒ | The exact sample size (*n*) for each experimental group/condition, given as a discrete number and unit of measurement |
| ☐ | ☒ | A statement on whether measurements were taken from distinct samples or whether the same sample was measured repeatedly |
| ☐ | ☒ | The statistical test(s) used AND whether they are one- or two-sided<br>*Only common tests should be described solely by name; describe more complex techniques in the Methods section.* |
| ☒ | ☐ | A description of all covariates tested |
| ☒ | ☐ | A description of any assumptions or corrections, such as tests of normality and adjustment for multiple comparisons |
| ☐ | ☒ | A full description of the statistical parameters including central tendency (e.g. means) or other basic estimates (e.g. regression coefficient) AND variation (e.g. standard deviation) or associated estimates of uncertainty (e.g. confidence intervals) |
| ☐ | ☒ | For null hypothesis testing, the test statistic (e.g. *F*, *t*, *r*) with confidence intervals, effect sizes, degrees of freedom and *P* value noted<br>*Give P values as exact values whenever suitable.* |
| ☒ | ☐ | For Bayesian analysis, information on the choice of priors and Markov chain Monte Carlo settings |
| ☒ | ☐ | For hierarchical and complex designs, identification of the appropriate level for tests and full reporting of outcomes |
| ☒ | ☐ | Estimates of effect sizes (e.g. Cohen's *d*, Pearson's *r*), indicating how they were calculated |

*Our web collection on statistics for biologists contains articles on many of the points above.*

## Software and code

Policy information about availability of computer code

| | |
|---|---|
| Data collection | Softwares Spectralis 6.8, Heidelberg Engineering, Germany; Diagnosys, Espion V6, Lowell, MA, USA. No custom code or algorithms were used. |
| Data analysis | Software SAS v9.4. No custom code or algorithms were used. |

For manuscripts utilizing custom algorithms or software that are central to the research but not yet described in published literature, software must be made available to editors and reviewers. We strongly encourage code deposition in a community repository (e.g. GitHub). See the Nature Research guidelines for submitting code & software for further information.

## Data

Policy information about availability of data

All manuscripts must include a data availability statement. This statement should provide the following information, where applicable:

- Accession codes, unique identifiers, or web links for publicly available datasets
- A list of figures that have associated raw data
- A description of any restrictions on data availability

The databases-approved official symbols used in this trial for CEP290 gene were HGNC, https://www.genenames.org/data/gene-symbol-report/#!/hgnc_id/HGNC:29021 and NCBI Gene, https://www.ncbi.nlm.nih.gov/gene/80184. Some data that support the findings of this study are available from ProQR Therapeutics. However, restrictions apply to the availability of these data, including intellectual property and confidentiality obligations, and so are not publicly available. Anonymized derived data will be made available by the authors upon reasonable request with methodologically sound proposal and with the permission of ProQR Therapeutics at hcp@proqr.com. All requests for raw and analyzed data are promptly reviewed, within a timeframe of 2 months, by a ProQR Therapeutics delegate and trial organizer, to verify if the request is subject to any intellectual property or confidentiality restrictions. Patient-related data not included in the paper were generated as part of clinical trials and may be subject to patient confidentiality. Any data that can be shared will be released via a data use agreement.

# Field-specific reporting

Please select the one below that is the best fit for your research. If you are not sure, read the appropriate sections before making your selection.

☒ Life sciences ☐ Behavioural & social sciences ☐ Ecological, evolutionary & environmental sciences

For a reference copy of the document with all sections, see nature.com/documents/nr-reporting-summary-flat.pdf

# Life sciences study design

All studies must disclose on these points even when the disclosure is negative.

| | |
|---|---|
| Sample size | The sample size of the study was not based on power calculations. It was selected based on clinical experience and considered to be adequate to fulfill the objectives of the study. For an ocular orphan disease, a sample size of approximately 12 patients was considered adequate to fulfill the safety objectives of the trial. |
| Data exclusions | It was found that two patients were mistakenly tested at some visits (screening until Month 2 for P7 and at Month 9 and Month 12 for P9) with a flash stimulus (duration <4 ms) instead of the pulse stimulus (duration, 200 ms) specified in the protocol. FST baseline data with the pulse stimulus test were missing for P7, so FST data for this patient were excluded from the efficacy analysis, whereas FST data at Month 9 and Month 12 were imputed for P9 by replicating Month 6 results. One patient was excluded from the mobility course analysis as baseline mobility course composite score was missing for this patient. Due to severe nystagmus in most of the patients, it proved to be very difficult to acquire images of suitable quality to be able to accurately measure all parameters with Spectral Domain Ocular Coherence Tomography (SD-OCT). |
| Replication | This is an early Phase first-in-man clinical trial and there was no replication |
| Randomization | The sample size of the study was not based on power calculations. It was selected based on clinical experience and considered to be adequate to fulfill the objectives of the study. For an ocular orphan disease, a sample size of approximately 12 patients was considered adequate to fulfill the safety objectives of the trial. |
| Blinding | This is a first-in-human open label trial. Masking was not possible as there was no sham-controlled procedure and the difference in response in treated eye versus untreated eye was obvious to both the patients and the investigators. |

# Reporting for specific materials, systems and methods

We require information from authors about some types of materials, experimental systems and methods used in many studies. Here, indicate whether each material, system or method listed is relevant to your study. If you are not sure if a list item applies to your research, read the appropriate section before selecting a response.

## Materials & experimental systems

| n/a | Involved in the study | |
|---|---|---|
| ☒ | ☐ | Antibodies |
| ☒ | ☐ | Eukaryotic cell lines |
| ☒ | ☐ | Palaeontology and archaeology |
| ☒ | ☐ | Animals and other organisms |
| ☐ | ☒ | Human research participants |
| ☐ | ☒ | Clinical data |
| ☒ | ☐ | Dual use research of concern |

## Methods

| n/a | Involved in the study | |
|---|---|---|
| ☒ | ☐ | ChIP-seq |
| ☒ | ☐ | Flow cytometry |
| ☒ | ☐ | MRI-based neuroimaging |

## Human research participants

Policy information about studies involving human research participants

| | |
|---|---|
| Population characteristics | The population ranged from 8 to 44 years of age. Five participants were male and 6 were female. All participants had a clinical diagnosis of LCA and a molecular diagnosis of homozygosity or compound heterozygosity for the CEP290 c.2991 +1655A>G/p.Cys998* allele. Six patients received loading/maintenace doses of sepofarsen of 160 µg/80 µg, and 5 received 320 µg/160 µg. |
| Recruitment | Individuals with a diagnosis of LCA10 due to the c.2991+1655A>G (p.Cys998X) mutation were recruited from the clinical practices and genetic registries of expert centers in the inherited retinal diseases field. The rarity of this specific orphan disease led to this recruitment process. Enrollment only occurred if inclusion criteria were met and the patient expressed interest in participation, given full discussion of the protocol and risks. It is unknown whether there was any self-selection bias, and how such a bias may have impacted the results. |
| Ethics oversight | The trial received Institutional Review Board/Ethics Committee approval of the University of Iowa Institute for Vision |

Ethics oversight

Research, the University of Pennsylvania and the Ghent University Hospital. The trial was conducted in accordance with the ethical principles of Good Clinical Practice and the Declaration of Helsinki. Adult participants provided written informed consent. Age-appropriate assent and permission from the parent or legal guardian were obtained for children.

Note that full information on the approval of the study protocol must also be provided in the manuscript.

# Clinical data

Policy information about clinical studies

All manuscripts should comply with the ICMJE guidelines for publication of clinical research and a completed CONSORT checklist must be included with all submissions.

Clinical trial registration

NCT03140969

Study protocol

The data that support the findings of this study are available from ProQR Therapeutics. However, restrictions apply to the availability of these data, including intellectual property and confidentiality obligations, and so are not publicly available. Data are, however, available from the authors upon reasonable request and with the permission of ProQR Therapeutics at hcp@proqr.com. All requests for raw and analyzed data are promptly reviewed by a ProQR Therapeutics delegate and trial organizer, to verify if the request is subject to any intellectual property or confidentiality restrictions. Patient-related data not included in the paper were generated as part of clinical trials and may be subject to patient confidentiality. Any data that can be shared will be released via a data use agreement.

Data collection

Patients were recruited between November 8, 2017, and September 27, 2018. Clinical data was collected at The University of Iowa Institute for Vision Research, University of Iowa, Iowa City, Iowa; The Department of Ophthalmology, Scheie Eye Institute, Perelman School of Medicine, University of Pennsylvania, Philadelphia, Pennsylvania and Ghent University Hospital, Ghent, Belgium between October 2017 to December 2019.

Outcomes

The primary end point was safety and tolerability of sepofarsen, assessed by frequency and severity of ocular adverse events in treated and untreated eyes at all study visits. Secondary end points included frequency and severity of non-ocular adverse events, serum pharmacokinetic profile of sepofarsen, changes in ophthalmic examination findings and changes in ophthalmic endpoints defined as standard methods to evaluate efficacy:
- Change from baseline in BCVA (assessed by the Early Treatment Diabetic Retinopathy Study [ETDRS] vision chart, or the Berkeley Rudimentary Vision Test for patients not able to read the letters on the ETDRS chart; Table S2),
- Change from baseline in dark-adapted retinal sensitivity using full-field stimulus test (FST) to red and blue stimuli,
- Anatomic measurements such as change from baseline in retinal structural evaluations using SD-OCT and NIRAF,
- Change in amplitude and latency to white pupillary light reflex (PLR).

Ophthalmic assessments were performed at all study visits (Table S3). An independent masked central reading center (EyeKor Inc.) was used to assess FST, SD-OCT and NIRAF-based end points. The serum pharmacokinetic profile of sepofarsen was assessed after IVT injection. Change from baseline in mobility course composite score was an exploratory end point and was interpreted and graded by a masked reader at Ora Inc. Additional exploratory end points included change in ocular instability; change in electroretinogram (ERG); change in Visual Function Questionnaire-25 (VFQ-25)/Cardiff Visual Ability Questionnaire for Children (CVAQC) score; change in biomarkers and further exploratory end points were included at the investigator's discretion (change in light sensitivity to white FST; change in amplitude/latency to red/blue PLR; change in outer segment layer thickness by OCT; visual evoked potential; standard FST; standard color vision test; visual field tests and additional light intensity tests on mobility course assessment). Further details on methodology are provided in the Supplemental Material.
